# Frequency-Coded Chipless RFID Tags: Notch Model, Detection, Angular Orientation, and Coverage Measurements

**DOI:** 10.3390/s20071843

**Published:** 2020-03-26

**Authors:** Jahangir Alam, Maher Khaliel, Abdelfattah Fawky, Ahmed El-Awamry, Thomas Kaiser

**Affiliations:** 1 Institute of Digital Signal Processing, Faculty of Engineering, University of Duisburg-Essen, 47057 Duisburg, Germany; maher.khaliel@id4us.de (M.K.); abdelfattah.megahed@id4us.de (A.F.); ahmed.elawamry@id4us.de (A.E.-A.); thomas.kaiser@uni-due.de (T.K.); 2 Benha Faculty of Engineering, Benha University, Benha 13511, Egypt

**Keywords:** chipless RFID, angular dependency, measurements

## Abstract

This paper focuses on the frequency coded chipless Radio Frequency Identification (RFID) wherein the tag’s information bits are physically encoded by the resonators’ notch position which has an effect on the frequency spectrum of the backscattered or retransmitted signal of the tag. In this regard, the notch analytical model is developed to consider the notch position and quality factor. Besides, the radar cross section (RCS) mathematical representation of the tag is introduced to consider the incident wave’s polarization and orientation angles. Hence, the influences of the incident wave’s orientation and polarization mismatches on the detection performance are quantified. After that, the tag measurement errors and limitations are comprehensively explained. Therefore, approaches to measureing RCS- and retransmission-based tags are introduced. Furthermore, the maximum reading range is theoretically calculated and practically verified considering the Federal Communications Commission (FCC) Ultra Wideband (UWB) regulations. In all simulations and experiments conducted, a mono-static configuration is considered, in which one antenna is utilized for transmission and reception.

## 1. Introduction

Recently, chipless Radio Frequency Identification has achieved a great importance in the research communities due to the tremendous possible applications [1,2,3]. However, the system is still at the conceptual level due to the challenges that are being faced to extract the tag-ID in real environments [4]. Since no Integrated Circuit is included, the tag-ID is physically realized either in the time or the frequency domain. Although time-coded tags have more detection robustness, frequency-coded (FC) tags are more practical in terms of coding capacity, tag size, and reader cost [5,6].

On the other hand, the chipped and chipless RFID technologies are identified using far field radio waves which operate at different frequency ranges, from low frequency (LF) to ultra-wide band (UWB) [7]. Near field communication (NFC) is a specialized subset within the family of RFID technology that uses a high frequency (HF), operating at a frequency of 13.56 MHz [8,9]. NFC devices must be in close proximity to each other, usually not more than a few centimetres (<0.1 m), and have data rates up to 424 kbps, whereas RFID has the ability to broadcast with a read range up to 20 m with a data rate of up to 640 kbps for the chipped one, as clarified in [7,10]. A brief comparison between NFC and chipless RFID is explained in Table 1.

The work introduced in this paper is focused on the FC chipless tags. The FC tags are classified into two main groups based on their structures. The first group constitutes radar cross section (RCS)-based tags, and the second group constitutes retransmission-based tags [11]. An RCS-based tag consists of a carrier substrate containing several scattering resonators for implementing the physical tag-ID. On the other hand, a retransmission tag contains two orthogonally-polarized antennas that are connected through a multi-stop band filter which realizes the tag-ID.

Of the state-of-the-art, several tag designs are available for both categories. They are orientation dependent, and most of them are polarization sensitive. The tag’s angular dependency has not been previously studied in the literature. Furthermore, the maximum detectable angular ranges are not elucidated from the simulation and measurement points of view. Consequently, the detection performance degradation is not quantified. Therefore, this paper aims to fill this gap, and also to present approaches to counteract the incident wave mismatches. In addition, the reader’s coverage is theoretically estimated and practically verified while considering the FCC regulations. However, in the literature, a certain reading range is normally reported without giving any further details about regulations.

The paper is organized as follows. The notch analytical model and the notch detection technique we utilized are introduced in Section 1. The influences of the incident wave’s polarization and orientation mismatches on the detection performance are illustrated in Section 2. In Section 3, the theoretical reading-range calculations are presented. The measurement errors and limitations are demonstrated in Section 4. The measurement results of the scenarios we investigated are introduced in Section 5. Concluding remarks are drawn in Section 6.

## 2. Notch Analytical Model and Notch Detection

In this section, the notch analytical model and the notch detection algorithm we applied are introduced.

### 2.1. Notch Analytical Model

The chipless RFID tag’s response consists of several notches in the frequency domain with predefined resonators, quality factors, and notching frequency positions. According to filter theory [12], the notch transfer function can be represented by zero and pole frequencies. The zero angular frequency, $\omega_{z},$ is represented by the resonance angular frequency, i.e., $\omega_{z}=\omega_{r}$ which is calculated using Equation (5); and the pole angular frequency, $\omega_{p}$, is represented by non-notch position. The zero frequency and pole frequency determine whether the response is standard, low pass, or high pass notch. The filter transfer function is given by,

(1a) $$H\left(i\omega \right)=\frac{\omega_{z}^{2}-\omega^{2}}{\left(\omega_{p}^{2}-\omega^{2}\right)+\left(\frac{\omega_{p}}{Q_{n}}\omega \right)i}$$

(1b) $$H\left(i2\pi f\right)=\frac{f_{z}^{2}-f^{2}}{\left(f_{p}^{2}-f^{2}\right)+\left(\frac{f_{p}}{Q_{n}}f\right)i}$$

where H(i2πf) is the filter transfer function; $\omega_{z}$ is the zero frequency, which is equal to $2\pi f_{z}$; $\omega_{p}$ is the pole frequency, which is equal to $2\pi f_{p}$; and $Q_{n}$ is the quality factor of the notch resonator.

The notch bandwidth $B_{n}$ of the resonator is related to the $Q_{n}$ factor and the notch position given in Equation (2).

(2) $$B_{n}=\frac{f_{z}}{Q_{n}}$$

Similarly, the notch pattern could be expressed as a function of frequency location $f_{z}$ and the corresponding resonator quality factor $Q_{n}$. Therefore, the notch pattern could be modeled as a 2nd order notch filter, as described in Equation (3). However, for standard notch, the zero and pole frequencies are equal, so the equation can be further modified as,

(3) $$H\left(i2\pi f\right)=\frac{f_{z}^{2}-f^{2}}{\left(f_{p}^{2}-f^{2}\right)+\left(\frac{f_{p}}{f_{z}}B_{n}\times f\right)i}$$

Thus, the notch pattern H(i2πf) could be expressed based on the notch bandwidth $B_{n}$ and the frequency position $f_{z}$.

Consequently, the notch is modulated by a specific bandwidth at a certain frequency location to codify a predetermined code (bitstream) $C_{k}$. Thereafter, the chipless tag response could be analytically described by Equation (4).

(4) $$\Gamma \left(i2\pi f\right)=\sum_{k=1}^{K}C_{k}{\cdot H\left(f\right)|}_{\left(f_{z},B_{n}\right)}$$

The chipless tag’s response Γ(i2πf) is analytically described based on the specified frequency position $f_{z}$ and the notch bandwidth $B_{n}$. Additionally, $C_{k}$ is the code factor that shows the presence or absence of the notch pattern at a certain frequency point. Consider the simplest case, which is binary coding, where a bit 0 or 1 is encoded by the absence or presence of the notch, respectively. In this regard, we nominate the notch absence to be the logic-0 or code-0. In order to validate the proposed notch model for FC chipless tags, a model is developed for two tag types with different quality factor resonators and encoding techniques.

**Figure 1:**
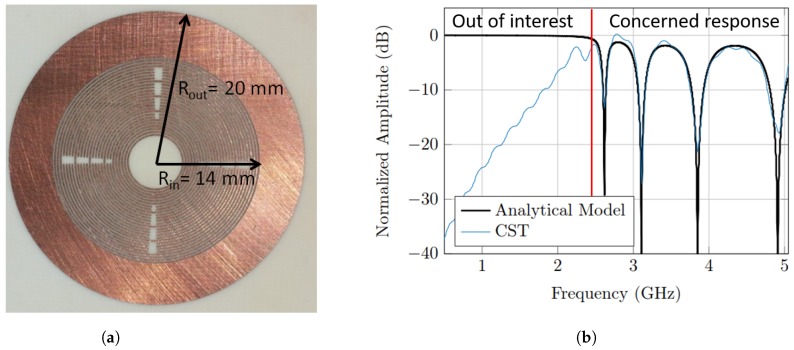
(**a**) Notch position modulation (NPM) radar cross section (RCS)-based tag. (**b**) Comparison between analytical results of the notch model and RCS simulation results obtained from CST for the tag which is based on the circular ring slot resonator.

The first one is the orientation independent tag represented in [13], wherein the coding element is the circular ring slot resonator with a quality factor of ≈20; the dimensions of unit cell are shown in Figure 1a, and the tag consists of 2×2 cells. The tag has been fabricated on RO4003C material with substrate thickness of 1.52 mm. The tag was made of coplanar ring resonators—width of 0.2 mm and gap of 0.2 mm, without a ground plane, so it could be printed. Each ring resonator represents a single bit and the resonance frequency for that corresponding ring is calculated by Equation (5).

(5) $$f_{r}=\frac{c}{2\pi R}\sqrt{\frac{2}{\epsilon_{reff}+1}}$$

where $f_{r}$ is the resonance frequency of the corresponding ring, *R* is the radius of the ring, *c* is the speed of light, and $\epsilon_{reff}$ is the effective permittivity of the substrate.

The simulated RCS response obtained from CST agrees very well with the model results, as shown in Figure 1b; the corresponding data of the analytical model are shown in Table 2. The low frequency region below the first notch in the developed model is not of interest, since it does not carry any coded information, as explained in Figure 1b. In radar theory, this out-of-interest region is called a Rayleigh region, in which the tag’s dimensions are considered to be much smaller than the wavelength. However, the concerned response is defined to be the resonance region in which the tag’s dimensions are comparable to the wavelength. Therefore, the RCS frequency response could reach minimum (notches) and maximum (peaks) values based on the engineered tag structure.

**Figure 2:**
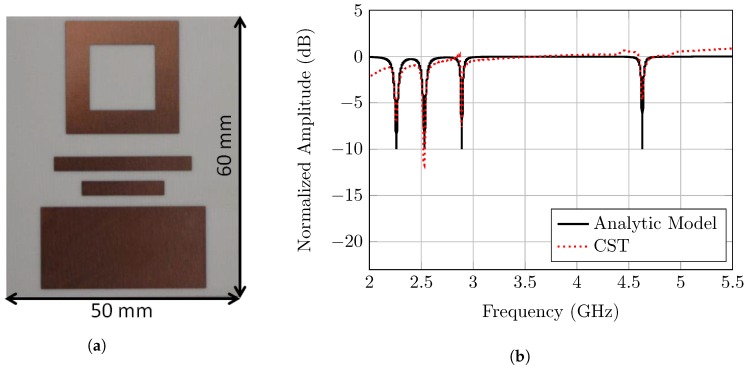
(**a**) Notch width modulation (NWM) RCS-based tag. (**b**) Comparison between analytical results of the notch model and RCS simulation results obtained from CST for the notch width modulation tag.

After that, the notch width modulation (NWM) tag, shown in Figure 2a and represented in [14], is utilized to complete the validation of the proposed notch model. The basic idea of the employed tag is to increase the coding capacity by utilizing different notch widths. Thus, the tag structure consists of three different quality factor resonators, which are the patch, the square ring, and the dipole with quality factors equal to 55, 80, and 100 respectively. The corresponding analytical data are listed in Table 3.

The tag-ID is configured to have the widest notch at the lowest frequency position, and the narrowest notch at the highest one, as shown in Figure 2b. This anti-logic order of the notches is to validate the robustness of the notch width modulation scheme and the corresponding notch model. Therefore, the chipless tag is designed and simulated for the RCS pattern with CST. The results which are presented in Figure 2b show a close match between the newly developed analytical notch model and the CST simulation results for various chipless tag resonators. Therefore, for any resonator type, the notch pattern and the information bits could be easily and quickly generated by utilizing the newly developed model. Hence, the channel’s influence on the notch pattern and also on the detection performance can be evaluated for the various tag-IDs.

### 2.2. Notch Detection

Notch detection algorithms are used to determine the existence of a notch in the backscattered signal from the tag. The signal processing involved in the notch detection is quite challenging, since the received signal power is very weak and is dramatically affected by undesired interference, environmental clutter, and noise caused by the receiver. Furthermore, manufacturing defects on the tag’s side may cause the notch to shift its frequency position or even change its shape. Another factor is the transmitting and/or the receiving antenna frequency response, which is superimposed on the received signal, as this also could modify the notch shape. All those factors could render the FC chipless RFID tag undetectable.

In this work, a matched filter notch detection technique is implemented to detect the ID of the interrogated tag. This will show the effects of different tag displacements, angular and range-related, on the detection probability of the tag notches. Matched filter is an ideal filter which calculates the maximum SNR (signal-to-noise ratio) of a received signal in the presence of additive stochastic noise [15]. It is commonly used in RADAR [16,17] and digital communication systems [18], where the original waveform is known, and the objective is to detect the presence of this signal against the background noise or distortion.

The algorithm works in three stages: The first stage is to obtain the matched filter response based on the RCS response of the tag. In each frequency range where a notch could exist, a matched filter response is created. The second stage is windowing the received signal and convoluting it with its respective predetermined matched filter. A decision is then made in the third stage, based on a predetermined threshold level.

The modified received signal R(f) can be obtained by adding wide-sense stationary random process D(f) to the received signal X(f) to represent the distortion caused by system imperfections, such as noise. The windowing function $W_{n}\left(f\right)$ is determined by first obtaining a received signal R(f) when the tag is at a predetermined position. Usually, at this position is where the tag is known to be at its best. The received signal is windowed in the frequency domain by multiplying with rectangular function, as illustrated in Equation (6).

(6) $$W_{n}\left(f\right)=R\left(f\right)\cdot rect\left(\frac{k}{f_{\max}-f_{\min}}-k\cdot f_{zn}\right)\;\;\;\;\mathrm{where}\;n=1,\ldots ,N$$

where *k* is number of samples in the window; *N* is total number of windows, which also corresponds to the number of notches; $f_{\max}-f_{\min}$ is the bandwidth of the window; and $f_{z}$ is the resonance frequency. The output of matched filter $Y_{n}\left(f\right)$ will be the convolution between the windowed signal $W_{n}\left(f\right)$ and matched filter function $H_{n}\left(f\right)$, as shown in Equation (7).

(7) $$Y_{n}\left(f\right)=H_{n}\left(f\right)*W_{n}\left(f\right)\;\;\;\;\;\mathrm{where}\;n=1,\ldots ,N$$

**Figure 3:**
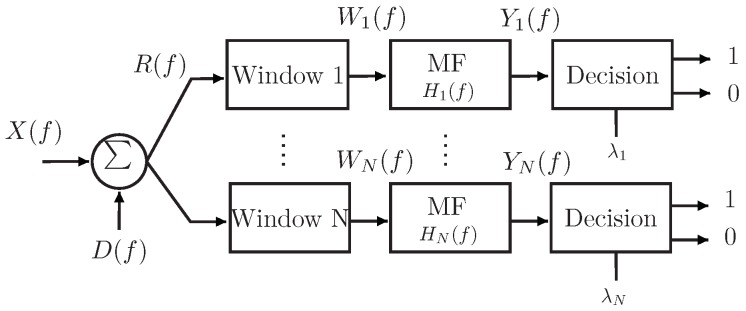
Matched filter implementation.

A window-based decision is performed at the end of $Y_{n}\left(f\right)$, which is based on a threshold $\lambda_{n}$ to determine whether a notch exists—“1” or not “0”—at a given window, as illustrated in Figure 3. The threshold is calculated by the Neyman–Pearson theorem of detection.

To evaluate the performance of the notch detection in various tag angular displacement scenarios, a probability of detection $P_{d}$ vs. signal-to-distortion ratio (SDR) will be plotted, where $P_{d}$ is given as follows:

(8) $$P_{d}=\frac{\mathrm{Correctly}\mathrm{detechted}\mathrm{notches}}{\mathrm{Total}\mathrm{number}\mathrm{of}\mathrm{notches}}$$

## 3. Angular Displacement

In the preceding studies [19,20,21,22,23,24,25], a perfect alignment between the reader antenna and the tag is assumed when simulating and measuring the tag response. However, the practical situation is different, for the tag’s azimuth and elevation angles are unknown to the reader antenna. Furthermore, tags that are rotation sensitive could be polarization mismatched with the interrogating antenna. These effects severely affect the tag’s response. Therefore, in this section the tag angular displacement problem is investigated, to accurately predict the tag responses for such scenarios.

### 3.1. Elevation Orientation Displacement

**Figure 4:**
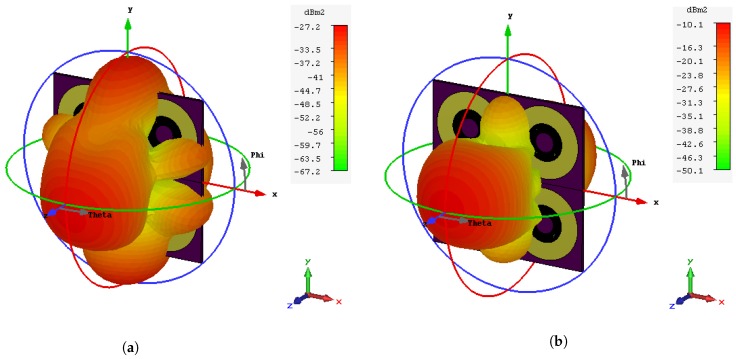
(**a**) 3-D RCS radiation pattern of the notch at 3.85 GHz and the tag structure. (**b**) 3-D RCS radiation pattern of the peak at 3.5GHz and the tag structure.

The simulation setup, as shown in Figure 4a, considers the tag which is presented in [13]. However, the setup is also valid for any other tag type. The angles ($\theta_{v},\theta_{h}$) represent the tag orientation in the vertical and horizontal directions respectively, as illustrated in Figure 4a. The reference configuration is considered to be the perfect alignment, where both transmitting and receiving antennas are pointing to the center of the tag. The 3-D RCS angular gain of the mono-static setup exhibits the tag directional radiation pattern for notched and non-notched frequencies, as illustrated in Figure 4b. It is worth noting that the notch and the peak radiation patterns are semi-symmetrical in both the vertical and horizontal theta planes. Figure 4a,b show RCS radiation patterns of a notch and peak respectively, for the tag which is based on the circular ring slot resonator.

**Figure 5:**
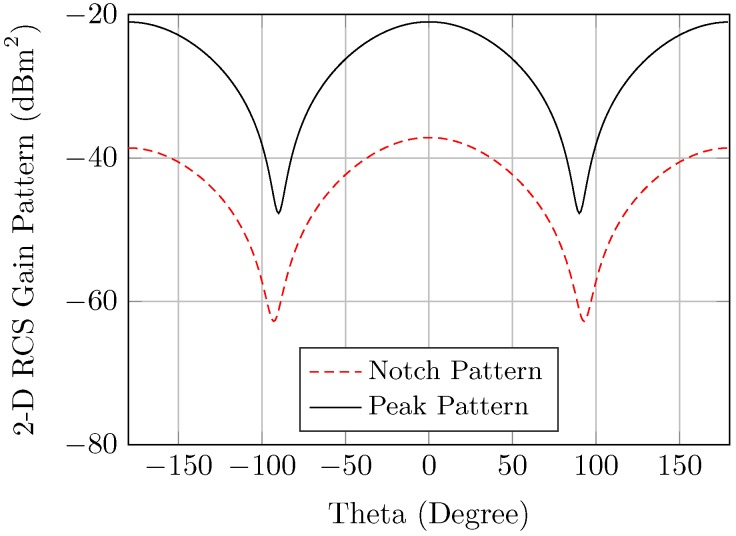
FC tag angular 2-D RCS patterns $\left(\theta_{v}=0,\theta_{h}=0\right)$ of notched and non-notched frequencies for the tag which is based on the circular ring slot resonator.

**Figure 6:**
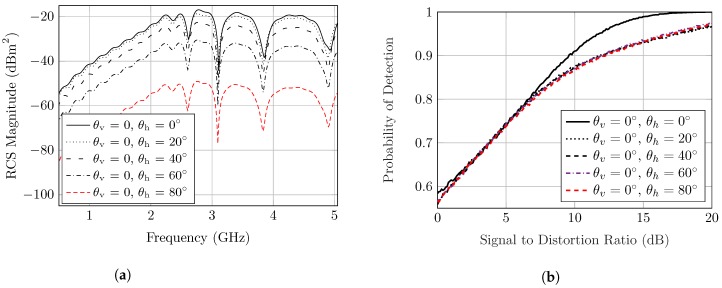
(**a**) Simulated RCS response of the polarization-independent circular slot ring’s tag with different incidence of observation angles $(\theta_{v}=0,\theta_{h}$). (**b**) Probability of detection for RCS response $(\theta_{v}=0,\theta_{h}$).

Therefore, the RCS fluctuations due to the variation of the elevation aspect angles could be anticipated from the 2-D radiation patterns, as described in Figure 5. Consequently, the tag’s RCS frequency response is varied in accordance with the gain of the radiation patterns, as explained in Figure 6a. As noticed, the worst decrease in the RCS values is about −15dBm when the observation angle is at $\theta_{h}$ = 90°. The same value could be observed for the variation of $\theta_{v}$. Figure 6b shows the $P_{d}$ of the angular variation of the notch and peak frequencies. This figure shows that the angular variation in this tag will just decrease the probability of detection by 0.05 at low distortion. At high distortion, they act very similarly.

### 3.2. Azimuth Orientation Displacement

In this subsection, the effect of tag rotation in the azimuth plane is investigated. This displacement causes a polarization mismatch loss, especially when the tag and reader antennas are linearly polarized. The linear polarization is mostly utilized to increase the reading range, in which the tag’s rotation angle is assumed to be fixed and known to the reader antenna. However, this is not a practical case.

**Figure 7:**
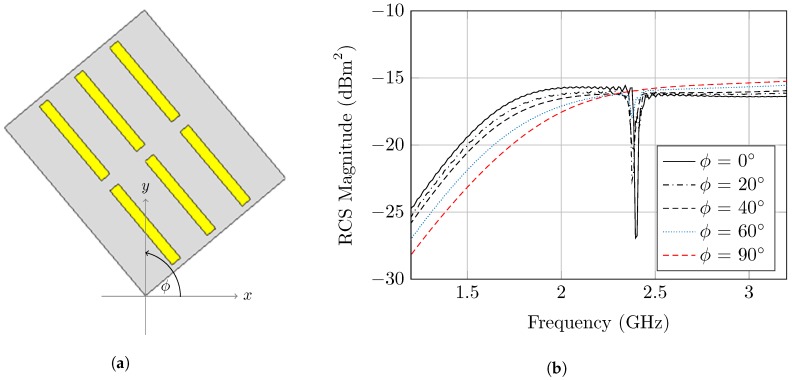
(**a**) Polarization mismatch simulation setup. (**b**) Simulated RCS frequency response for the polarization-dependent dipole based tag at five different orientation angles (0°, 20°, 40°, 60°, 90°).

Hence, the polarization mismatch is investigated, where the rotation angle around the tag axis is considered to be ϕ as clarified in Figure 7a. The previously introduced tag with circular rings is polarization-independent, and thus a linear polarizing tag such as the one introduced in [26] is designed to be utilized in the study. However, the study is also applicable to any polarization-dependent tag.

Therefore, a linear, polarized dipole resonator is utilized for the study, and the corresponding co-polarized and cross-polarized RCS responses are observed. Basically, the notch dynamic range diminishes in the co-polarized plane with the increasing of the tag rotation angle.

However, the notch could be detected up to ϕ = 60°, where the notch depth is reduced to 2dB. However, the notch completely vanishes at ϕ = 90°. In this case, the polarization is altered, and thus the cross polarized component has a significant effect which alters the co-polarized response.

Figure 7b shows that altering ϕ will change the detection capability of tag; ϕ = 20° has a similar performance to the angle ϕ = 0°. At 40° and 60° the $P_{d}$ was degraded severely. The angle ϕ = 90° was omitted since it was not detected for all values of the SDR.

It is clearly noticed that the observed backscattering area depends on the angular orientation of the tag, relative to the reader antenna. Hence, the 3-D RCS angular gain should be taken into consideration in order to have accurate RCS calculations. Moreover, a slight frequency shift could be produced from a change of the tag apparent size, as shown in Figure 7b.

## 4. Reading Range

In this section, the theoretical reading range of the FC chipless tag is calculated. Therefore, the corresponding maximum detectable ranges are presented. The general range equation for the RCS tags can be expressed as [27] Equation (9), considering the mono-static configuration.

(9) $$r_{\mathrm{range},\;\mathrm{RCS}-\mathrm{tags}}=\sqrt[{4}]{\frac{G_{R}^{2}\lambda^{2}P_{T}}{{\left(4\pi \right)}^{3}P_{\min}}\sigma \left(f,\theta ,\phi \right)}$$

where $r_{\mathrm{range},\;\mathrm{RCS}-\mathrm{tags}}$ is the reading range for the RCS based tags, $G_{R}$ is the reader antenna gain, λ is the wavelength, $P_{T}$ is the transmitted power, $P_{\min}$ is the reader sensitivity, and σ(f,θ,ϕ) is the RCS tag’s response.

Regarding the retransmission tag, the tag consists of two orthogonally polarized antennas and a notch filter for realizing the tag-ID. Therefore, the reading range equation is modified to consider the transmitting and receiving antenna gains of the tag, and the insertion loss of the filter as well. Considering that the transmitting and receiving antennas of the tag are the same, the reading range could be expressed as in Equation (10).

(10) $$r_{\mathrm{range},\;\mathrm{retrans}.-\mathrm{tags}}=\sqrt[{4}]{\frac{G_{R}^{2}G_{T}^{2}\lambda^{4}P_{T}}{{\left(4\pi \right)}^{4}P_{\min}}IL^{4}\left(f\right)}$$

where $G_{T}$ is the tag antenna gain, and IL(f) is the filter insertion loss which varies with frequency.

**Figure 8:**
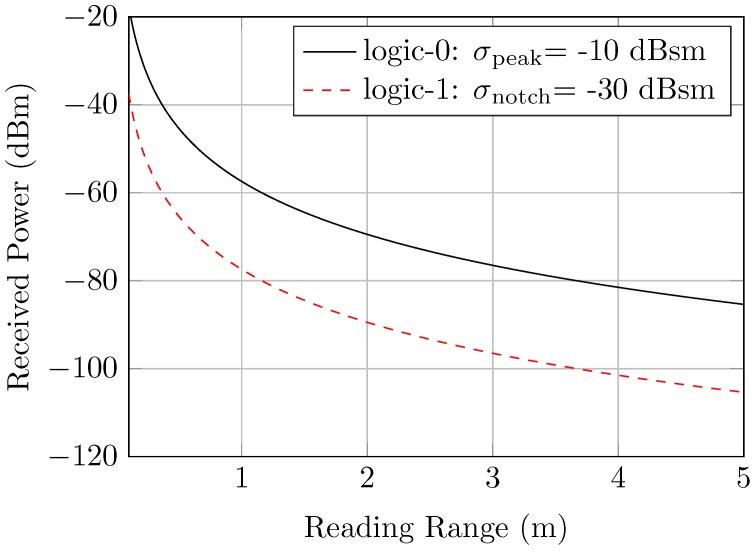
The theoretical range calculations of the RCS tags.

For both tag types, the reading methodology which is presented in [28] is utilized. This methodology is applied to the RCS tags where the RCS value of the peak is considered to be −10dBsm, and the RCS value of the notch is −30dBsm. Therefore, the theoretical estimation of the range is illustrated in Figure 8, assuming that the reader antenna gain $G_{R}=10\;\mathrm{dBi}$, and the maximum Effective Isotropic Radiated Power (EIRP) is equal to 0dBm/50MHz.

Consider that the receiver sensitivity is –80 dBm. As a consequence, the maximum theoretical reading range for the RCS tag will be 3 m, in which the notch dynamic range is 3 dB, as shown in Figure 8. However, the practical reading range is expected to be slightly lower due to the realistic considerations which will be discussed in the next sections.

## 5. FC Tags: Measurement Errors and Limitations

This section describes the measurement errors which limit chipless tag detection. Basically, the measurement errors are produced from the amplitude phase statistical noise and from the reader systematic errors. The statistical noise could be reduced by averaging over the several measurement cycles. Moreover, the systematic error could be modeled to calculate the tag’s frequency response accurately [29].

Fundamentally, the first systematic error is the component matching, which is not uniform with frequency, like the antenna matching with couplers, switches, and so on. The second major limitation is the reader’s circuit leakage channels. The third measuring error appears from the nonlinear circuit of the reader and the antenna’s finite polarization purity. Therefore, the minimization of these realistic imperfections is of the utmost import when in comes to accurately measuring the RCS tag and enhancing the reader’s coverage.

**Figure 9:**
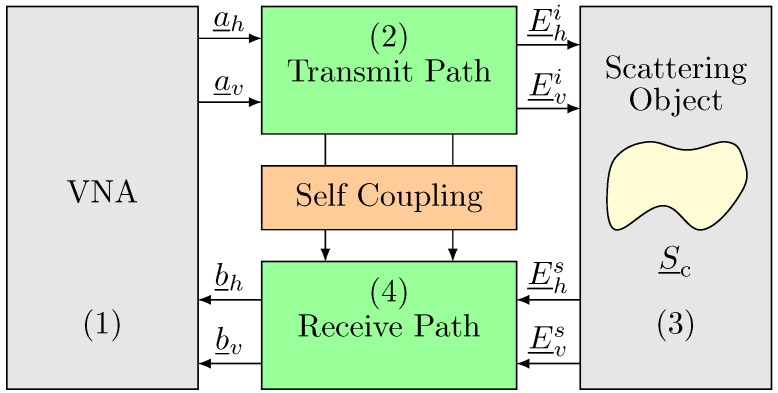
Measurement setup model.

In this regard, the mathematical models of these error terms are introduced. Hence, the reader functions are mathematically described in Figure 9, where the tag scattering matrix is related to the complex RCS matrix, as defined in Equation (11).

(11) $${\underline{S}}_{c}=\frac{1}{\sqrt{4\pi r^{2}}}\sqrt{\underline{\sigma }}$$

where $\underline{\sigma }$ is the RCS matrix.

The Vector Network Analyzer reader is equipped in the first block; the transmitter path is represented by the second block; the tag inscribed metallic scatterer is represented by the third block; and lastly, the receiver path is represented in the fourth block, as described in Figure 9. The VNA is capable of measuring the transmitting $\left({\underline{a}}_{v},{\underline{a}}_{h}\right)$ and receiving signals $\left({\underline{b}}_{v},{\underline{b}}_{h}\right)$ as well.

Therefore, the measured scattering matrix could be calculated from these transmitted and received signals based on Equation (12).

(12) $$\left[\begin{array}{c} {\underline{b}}_{h}\\ {\underline{b}}_{v} \end{array}\right]=\left[\begin{array}{cc} {\underline{S}}_{hh,m} & {\underline{S}}_{hv,m}\\ {\underline{S}}_{vh,m} & {\underline{S}}_{vv,m} \end{array}\right]\left[\begin{array}{c} {\underline{a}}_{h}\\ {\underline{a}}_{v} \end{array}\right]$$

The measured scattering matrix is related to the tag correct scattering matrix ${\underline{S}}_{c}$ by Equation (13).

(13) $${\underline{S}}_{m}=\underline{I}+\underline{R}\cdot {\underline{S}}_{c}\cdot \underline{T}$$

where ${\underline{S}}_{c}$ is the correct scattering matrix of the tag, which is erroneous by the additive coupling matrix, and the multiplicative transmission $\underline{T}$ and reception $\underline{R}$ matrices, as explained in Equation (14).

(14) $$\begin{array}{c} \left[\begin{array}{cc} {\underline{S}}_{hh,m} & {\underline{S}}_{hv,m}\\ {\underline{S}}_{vh,m} & {\underline{S}}_{vv,m} \end{array}\right]=\left[\begin{array}{cc} {\underline{I}}_{hh} & {\underline{I}}_{hv}\\ {\underline{I}}_{vh} & {\underline{I}}_{vv} \end{array}\right]+\left[\begin{array}{cc} {\underline{R}}_{hh} & {\underline{R}}_{hv}\\ {\underline{R}}_{vh} & {\underline{R}}_{vv} \end{array}\right]\cdot \left[\begin{array}{cc} {\underline{S}}_{hh,c} & {\underline{S}}_{hv,c}\\ {\underline{S}}_{vh,c} & {\underline{S}}_{vv,c} \end{array}\right]\cdot \left[\begin{array}{cc} {\underline{T}}_{hh} & {\underline{T}}_{hv}\\ {\underline{T}}_{vh} & {\underline{T}}_{vv} \end{array}\right] \end{array}$$

Hence, the correct scattering matrix ${\underline{S}}_{c}$ is subject to twelve error components, which are isolation, transmission, and reception errors [29,30]. If these error-terms are known, the correct scattering matrix could be calculated by inversion, as explained in Equation (15).

(15) $${\underline{S}}_{c}={\underline{R}}^{-1}\cdot \left\{{\underline{S}}_{m}-\underline{I}\right\}\cdot {\underline{T}}^{-1}$$

The unknown error terms could be calculated from the measurement of reference targets with known scattering matrix ${\underline{S}}_{c}$ [30]. Thus, the additive error coefficients $\underline{I}$ are calculated first from the measurement of empty room calibration, where ${\underline{S}}_{c}\approx 0$. Then, these isolation terms are known as indicated $\underline{I}={\underline{S}}_{m}$. Consequently, the measured scattering matrix could be rewritten as in Equation (16).

(16) $${\underline{S}}_{m}-\underline{I}={\underline{S}}_{\mathrm{mi}}=\underline{R}\cdot {\underline{S}}_{c}\cdot \underline{T}$$

The goal now is to determine these multiplicative error terms. Therefore, reference targets with known RCS could be utilized for calculating the multiplicative error terms. Therefore, a single reference algorithm is utilized for calculating the error coefficients with the knowledge of the scattering matrix of one target. The algorithm assumes that the cross-polarization signals are negligible (e.g., spheres and circular disks).

Hence, the correct RCS tag could be calculated by utilizing one known reference target measurement besides the empty room calibration. However for the retransmission tags, these assumptions are not valid, since the tag response is configured in the cross-polarized plane. Therefore, the depolarizing error terms should be considered. In this regard, a reference depolarizing tag is designed to calibrate the measurements of the retransmission tag.

## 6. FC Tags Measurements

In this section the experimental results for the previously discussed scenarios are introduced. Therefore, the calibration procedures for both RCS and depolarizing tags are presented first. Then, the angular displacement measurements are demonstrated. Finally, the maximum reading range was experimentally determined, while considering the FCC regulations.

### 6.1. RCS and Depolarizing Tags’ Measurements

**Figure 10:**
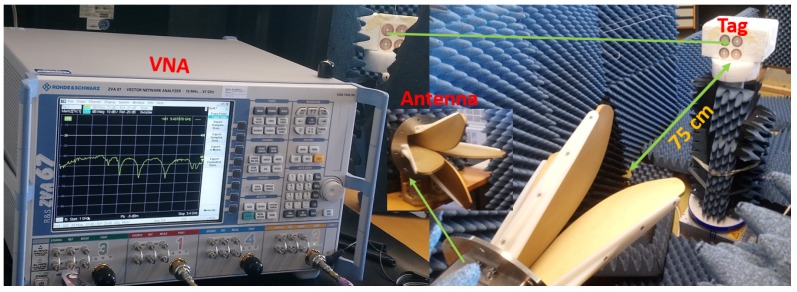
Measurement setup.

The mono-static measurement setup can be seen in Figure 10. The VNA is connected to a dual polarized horn antenna with 10 dBi gain at 5 GHz. For measuring the orientation dependency of the RCS tags, the circular ring tag is manufactured and placed on a rotating rod as shown in Figure 10.

We followed the calibration procedures which are described in Section 4, and considered only one polarization channel, since the circular ring tag does not alter the polarization of incident wave. Therefore, the measured scattering matrix is simplified in Equation (17).

(17) $${\underline{S}}_{hh,m}={\underline{I}}_{hh}+{\underline{R}}_{hh}\cdot {\underline{S}}_{hh,c}\cdot {\underline{T}}_{hh}$$

Utilizing the empty room calibration the measured backscattered reflection is expressed in Equation (18).

(18) $${\underline{S}}_{hh,m}-{\underline{I}}_{hh}={\underline{S}}_{hh,\mathrm{mi}}={\underline{R}}_{hh}\cdot {\underline{S}}_{hh,c}\cdot {\underline{T}}_{hh}$$

**Figure 11:**
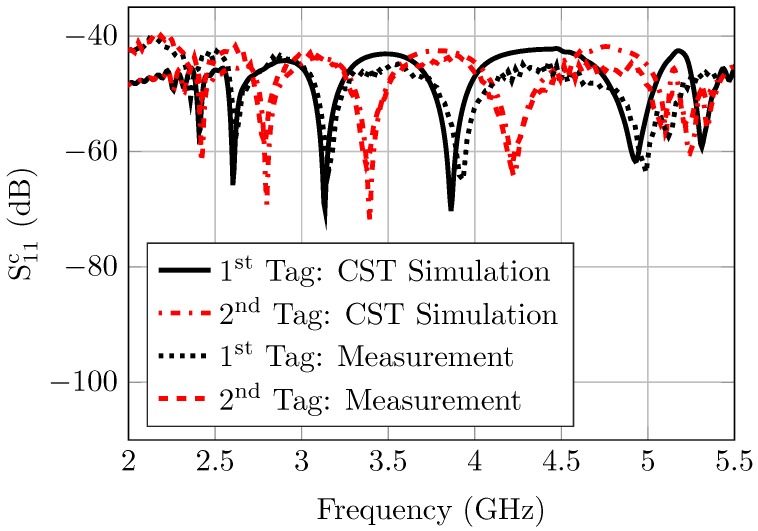
Calibrated measured reflection coefficients of two different codes for the circular ring slot tag.

Hence, a perfect conducting sphere of 15mm diameter is utilized to calculate the multiplicative error terms. Therefore, the magnitude spectrum of the correct reflection coefficient is measured for two different codes to verify the calibration procedures. The notches represented in Figure 11 are for two different tags which we have simulated and measured. The notches of the first tag represent (1101010101) code while the notches for the second tag represent (1010101010). These results indicate that the simulations and the measurements are well matched. However, a frequency shift was observed in the measurement results. It occurred due to the fabrication tolerance. It can also occur when the calibration of a system’s integration is not ideal.

**Figure 12:**
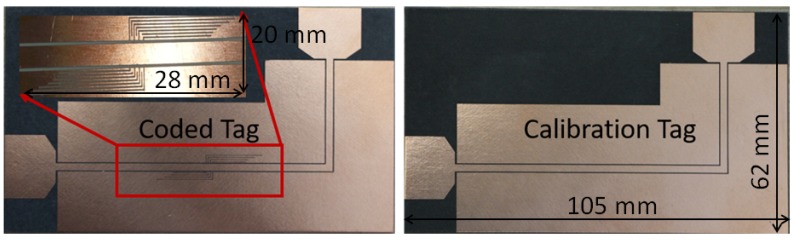
Depolarizing coded tag on left and the corresponding calibration tag on right.

Regarding depolarizing error terms, a reference tag is designed to be used for calibration. The calibration tag consists of two orthogonally polarized antennas which are connected through a coplanar transmission line. On the other hand, for the coded tag, the two antennas are connected through a multi-stop band filter that realizes the tag-ID as shown in Figure 12.

For measuring the retransmission tags, the two orthogonally polarized channels of the horn antenna are utilized. The cross polarization isolation is more than 30 dB, which is the highest possible with single antenna. Consequently, the reader non-ideal depolarizing terms are much lower than the tag response after doing the empty room calibration. Considering that the incident wave is horizontally polarized, the tag response is configured in the vertical polarization plane and the measured scattering matrix is simplified in Equation (19).

(19) $${\underline{S}}_{vh,m}={\underline{I}}_{vh}+{\underline{R}}_{vv}\cdot {\underline{S}}_{vh,c}\cdot {\underline{T}}_{hh}$$

Utilizing the empty room calibration, the measured depolarized retransmission is expressed in Equation (20).

(20) $${\underline{S}}_{vh,m}-{\underline{I}}_{vh}={\underline{S}}_{vh,\mathrm{mi}}={\underline{R}}_{vv}\cdot {\underline{S}}_{vh,c}\cdot {\underline{T}}_{hh}$$

After that, the calibration tag is utilized for calculating the multiplicative error terms, as explained in Equation (21).

(21) $${\underline{R}}_{vv}\cdot {\underline{T}}_{hh}={\underline{S}}_{vh,\mathrm{mi}}\cdot {\underline{S}}_{vh,c}^{-1}$$

**Figure 13:**
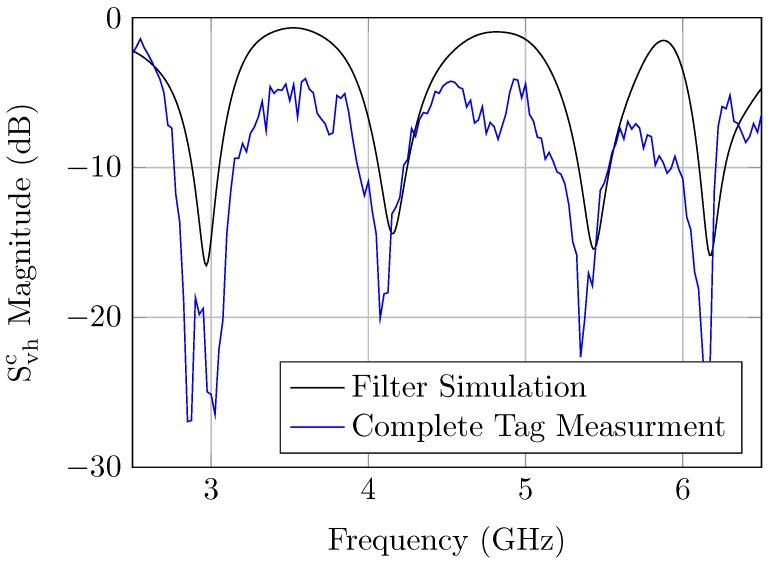
Depolarizing retransmission tag measurement.

Therefore, the relevant depolarizing errors of the tag antennas are minimized, and the retransmitted response of the tag-ID can be detected. The calibrated measured tag response is shown in Figure 13. The disturbances in the whole-tag measurements resulted from the fabrication tolerance, the filter insertion loss, and the mismatch of the antennas.

### 6.2. Elevation Orientation Displacement Measurement

**Figure 14:**
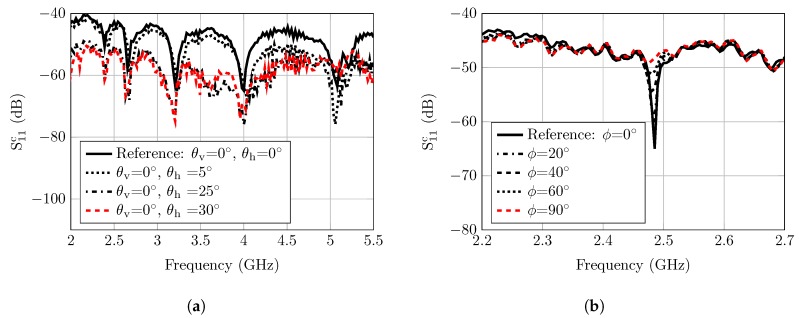
(**a**) Calibrated reflection coefficients of different displacement angles in the elevation plane for the tag which is based on the circular slot ring resonators. (**b**) Calibrated reflection coefficients for different displacement angles in the azimuth plane.

The angular displacement in the elevation plane is measured at three different angles, as explained in Figure 14a. The measured RCS response indicates that the notch level and pattern are highly degraded with the increase of the misalignment angle (especially the higher frequency notches). Moreover, the notch at 5.2 GHz is completely distorted if the elevation displacement exceeds 30°. One possible solution with which to mitigate this problem is to enable retro-directivity at the tag side, which is very hard to achieve with planar structure over a wide range of frequencies.

### 6.3. Azimuth Orientation Displacement Measurement

As previously mentioned, the azimuth displacement produces a polarization mismatch between the tag resonators and reader antenna. Therefore, a linear polarized tag was designed and manufactured to practically assess the performance degradation due to polarization mismatching. The tag consisted of half wavelength dipoles which resonated at 2.48GHz. The reader setup was the same as before, and the tag was rotated to implement the polarization mismatch. Moreover, the calibration procedures, as previously explained, were applied, and the measurement results are presented in Figure 14b.

The notch depth decreases with the increment of the polarization misalignment angle. However, the notch’s decrease in the co-polarized plane increases the notch level in the cross-polarized plane. It is concluded from these results that the elevation misalignment severely reduces the RCS value of the tag response, and this reduction is not uniform overall the tag response. The reason is that the higher frequency notches are exposed to more path loss. Therefore, the expected reading range is less. On the other hand, the azimuth misalignment only affects the notch’s dynamic level. Moreover the polarization mismatch problem could be easily solved by employing a dual-polarized antenna at the reader side, like the one that we are using. However, to mitigate the orientation mismatch problem, we have to employ more reading antennas around the tag interrogation zone.

### 6.4. Range Measurement

**Figure 15:**
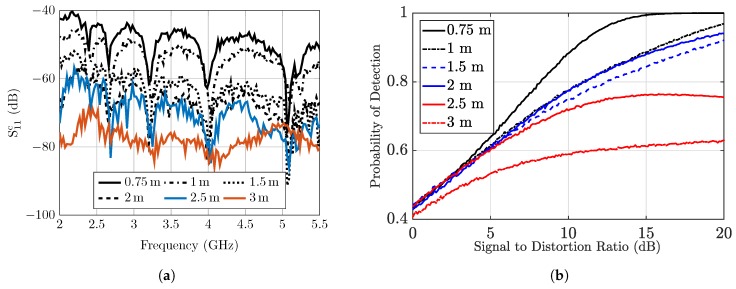
(**a**) Calibrated reflection coefficient measurement at different distances from the reader for the tag which is based on the circular ring slot resonator. (**b**) Probabilities of detection for different distances.

The previously described setup shown in Figure 10 is utilized for determining the maximum readable range. Therefore, the tag is placed at several distances from the reader until the tag response is completely undetectable. The measurement results indicate that the tag could be detected up to 2.5 m away from the reader antenna, as explained by Figure 15a.

The theoretical calculation of the noise floor of this setup is about −78dBm, and thus any signal below this value is not detected. Therefore, the 3m measurements are contaminated by the reader noise. Hence, the theoretical limits which are calculated in Section 3 are approached practically.

It is also clear from Figure 15b that as the tag distance from the reader increases $P_{d}$ as the notches degrade. In Figure 15b the signal power at a given distance is constant but the distortion is varying. The variation of the distortion may be caused by the changing of the channel clutter response or calibration inaccuracy. The curve also indicates that even at high distances (3m) the chances are higher than 60% that the notches will detected at high SDR.

## 7. Conclusions

In this paper, the major limitations of the FC chipless RFID tags are theoretically and practically investigated. The various tag designs which have been employed in this study are summarized in Table 4. A model is proposed to analytically describe the notch patterns of the different notch coding techniques. It yields a good match to the designed chipless tags using the CST-Microwave Studio EM simulator. This model helps in evaluating the channel’s influence on the information bits and also on the detection performances of the various tag-IDs easily and effectively. After that, the angular dependency of the tag’s response is introduced. In this regard, the tag’s radiation patterns for the notched and non-notched frequencies are considered. Consequently, the readable aspect angle’s range is demonstrated by simulation and verified by measurements. Moreover, a notch detection algorithm using matched filter is considered and implemented on different tag orientations. Lastly, the maximum reading range is theoretically calculated and practically measured.

## Figures and Tables

**Table 1:** Comparison between near field communication (NFC) and chipless RFID.

Parameters	NFC	Chipless RFID
Antenna Size	Bigger	Smaller
Operating Frequency	13.56 MHz	UWB
Transfer Efficiency	Lower(Nearfield Antenna)	Higher (Farfield Antenna)
Power Transfer	Depends on mutual coupling	Depends on the RCS of the chipless tag
Distance	<0.1 m	Can achieve 2 m and more

**Table 2:** The NPM tag’s analytical model data.

$f_{z}\;(\mathrm{GHz})$	$B_{n}\;(\mathrm{MHz})$	$Q_{n}$	$C_{k}$
2.618	112	23.750	1
3.108	266	11.6842	1
3.85	448	8.5938	1
4.91	592	8.2939	1

**Table 3:** The NWM tag’s analytical model data.

$f_{z}\;(\mathrm{GHz})$	$B_{n}\;(\mathrm{MHz})$	$Q_{n}$	$C_{k}$
2.26	41.0909	55	1
2.53	46	55	1
2.89	36.125	80	1
4.63	46.3	100	1

**Table 4:** Various tag designs—summary.

	Figure Number	Modulation (Coding)	Size (mm)	Resonator Type	Polarization	Coding Capacity
**RCS-based**	Figure 1a	Notch Postion	80×80	Ring	Independent	Moderate
	Figure 2a	Notch Postion and Width	50×66	Patch, square ring, and dipole	Linear	High
	Figure 7a	Notch Postion	60×50	Dipole	Linear	Low
**Retransmission-based**	Figure 12	Notch Postion	62×105	Slot	Dual	Low
